# Heart transplantation and biomarkers: a review about their usefulness in clinical practice

**DOI:** 10.3389/fcvm.2024.1336011

**Published:** 2024-01-24

**Authors:** L. Martini, G. E. Mandoli, M. C. Pastore, A. Pagliaro, S. Bernazzali, M. Maccherini, M. Henein, M. Cameli

**Affiliations:** ^1^ Department of Medical Biotechnology, University of Siena, Siena, Italy; ^2^ Cardio-Thoracic-Vascular Department, Siena University Hospital, Siena, Italy; ^3^ Department of Public Health and Clinical Medicine, Umeå University, Umeå, Sweden

**Keywords:** heart transplantation, biomarker, CAV, PGD, RVD, rejection

## Abstract

Advanced heart failure (AdvHF) can only be treated definitively by heart transplantation (HTx), yet problems such right ventricle dysfunction (RVD), rejection, cardiac allograft vasculopathy (CAV), and primary graft dysfunction (PGD) are linked to a poor prognosis. As a result, numerous biomarkers have been investigated in an effort to identify and prevent certain diseases sooner. We looked at both established biomarkers, such as NT-proBNP, hs-troponins, and pro-inflammatory cytokines, and newer ones, such as extracellular vesicles (EVs), donor specific antibodies (DSA), gene expression profile (GEP), donor-derived cell free DNA (dd-cfDNA), microRNA (miRNA), and soluble suppression of tumorigenicity 2 (sST2). These biomarkers are typically linked to complications from HTX. We also highlight the relationships between each biomarker and one or more problems, as well as their applicability in routine clinical practice.

## Introduction

Heart transplantation (HTx) is the definitive treatment for advanced heart failure (AdvHF). It reduces the mortality rate and improves the quality of life of patients (1). However, there are still several contraindications to heart transplantation, most of which are serious concomitant diseases such as severe peripheral disease or malignancies with a poor prognosis (Table 1) (2). According to Olmsted County, Minnesota, the availability of new drugs and devices and the introduction of new surgical techniques have led to an increase in the number of patients with AdvHF in AHA stage C from 93,600 to 124,800 and in AHA stage D from 15,600 to 156,000. Despite this, the number of heart donors is not increasing, resulting in a mismatch between supply and demand (3).

**Table 1:** Heart transplantation indications and contraindications according to the ESC guidelines (2).

Indications
Advanced heart failure
No other therapeutic option, except for LVAD as BTT
Contraindications
Active infections
Severe peripheral arterial or cerebrovascular disease
Pharmacologic irreversible pulmonary hypertension
Malignancy with poor prognosis
Irreversible liver dysfunction or irreversible renal dysfunction
Systemic disease with multiorgan involvement
Other serious comorbidity with poor prognosis
Pre-transplant BMI >35 kg/m2
Current alcohol or drug abuse
Psychological instability that jeopardizes proper follow-up and intensive therapeutic regime after heart transplantation
Insufficient social supports to achieve compliant care in the outpatient setting

For this reason, it has become necessary to draw up lists in which patients with AdvHF are stratified according to the severity of HF. One of the best-known criteria for such a classification is the US list allocation system, the United Network for Organ Sharing (UNOS) allocation criteria. It divides patients into six categories based on their clinical status, comorbidities and the use of mechanical support and/or positive inotropes. Patients with status 1 have a higher priority than patients with status 6, who have a lower priority (Table 2) (4).

**Table 2:** UNOS allocation criteria (4).

Status	Criteria
1	• VA-ECMO • Non-dischargeable, surgically implanted, non endovascular biventricular support device • MCSD with life-threatening ventricular arrhythmia
2	• IABP • Non-dischargeable, surgically implanted, non endovascular LVAD • VT or VF without mechanical support • MCSD with device malfunction or failure • TAH, BiVAD, RVAD, or VAD for single ventricle patients • Percutaneous endovascular MCSD
3	• Dischargeable LVAD for discretionary 30 days • Multiple inotropes or single high-dose inotrope with continuous hemodynamic monitoring • Single inotrope with continuous monitoring • VA-ECMO after 7 days; IABP or percutaneous endovascular circulatory support device after 14 days • Non-dischargeable, surgically implanted, non endovascular LVAD after 14 days • Mechanical support device with complication
4	• Dischargeable LVAD without discretionary 30 days • Inotropes without hemodynamic monitoring • Retransplant • Diagnosis of CHD, ischemic heart disease with intractable angina, hypertrophic CM, restrictive CM, amyloidosis
5	• On waitlist for at least one other organ at the same hospital
6	• All other active candidates

CHD, congenital heart disease; CM, cardiomyopathy; ECMO, veno-arterial extracorporeal membrane oxygenation; IABP, intra-aortic balloon pump; (R/l) VAD, (right/left) ventricular assist device; TAH, total artificial heart; VA-MCSD, mechanical circulatory support device; VF, ventricular fibrillation; VT, ventricular tachycardia.

After HTx, immunosuppressive drugs are usually used to protect against complications related to autoimmunity. To identify patients with early rejection, invasive procedures such as endomyocardial biopsy (EMB) and coronary angiography for vasculopathies are often required (5). Several studies have shown associations between several HTx complications and different types of biomarkers, emphasizing the useful utility of the latter. This review describes the association between biomarkers and HTx complications and analyses their unique and important use in clinical practice.

### Complications in heart transplantation

Primary graft dysfunction (PGD) is the number one cause of early mortality in HTx. The International Society of Heart and Lung Transplantation (ISHLT) defines PGD as a primary graft failure involving the left and/or right ventricle, with echocardiographic and hemodynamic changes requiring inotropic/vasopressor support and usually necessitating the use of circulatory support devices. The pathophysiology of PGD is not well understood, although an ischemia-reperfusion mechanism may be suspected. PGD usually manifests with hemodynamic instability and cardiogenic shock (5).

Right ventricular dysfunction (RVD) is another problem commonly caused by pulmonary hypertension (PH) in recipients. It often occurs in patients with an elevated pulmonary vascular resistance (PVR) greater than 4 WU, a systolic pulmonary arterial pressure (sPAP) greater than 60 mmHg and a transpulmonary gradient greater than 15 mmHg. Vasodilators for the pulmonary circulation and often positive inotropes are the treatment of choice for patients with RVD (5).

The occurrence of infections in HTx is a common complication due to the use of immunosuppressive drugs. In the first month, nosocomial infections are the most common infections, less severe are mucocutaneous candidiasis and zoster reactivations. In the second month of HTx, CMV, toxoplasmosis, aspergillosis and P. jirovecii infections are the most common pathogens. From the sixth month onwards, the aetiological pathogens no longer differ from those that occur in immunocompetent patients (5).

HTx rejection is usually divided into hyperacute, cellular and humoral rejection. Hyperacute rejection is due to pre-formed antibodies against donor antigens. Erythrocyte antigens of the AB0 group and HLA are the most common targets of these antibodies. In this type of rejection, it is extremely important to avoid incompatibilities between the transplant and the patient during the pre-transplant assessment. Cellular rejection is the most common form, characterized by the presence of inflammatory cells in the myocardium, usually T-cells and neutrophils. Finally, humoral rejection is caused by antibodies (usually anti-HLA) against the vascular endothelium and is usually associated with a poor outcome. Immunosuppressive therapy and plasmapheresis are the therapies used to combat these complications (5).

Cardiac allograft vasculopathy (CAV) is a late complication characterized by a persistent perivascular inflammatory state associated with intimal hyperplasia. It manifests clinically in the form of coronary syndromes and is the most important life-limiting factor. In these patients, coronary angiography is performed at regular intervals to assess the early development of coronary disease and its progression. Since the pattern of coronary atherosclerosis in these patients is diffuse, revascularization is often difficult, making re-transplantation the only definitive therapy (5).

Immunosuppression is also associated with the development of tumours. Non-Hodgkin lymphoma (NHL), Hodgkin lymphoma (HL), Kaposi sarcoma (KS), anogenital and hepatic tumours are the most commonly documented (5).

**Figure 1:**
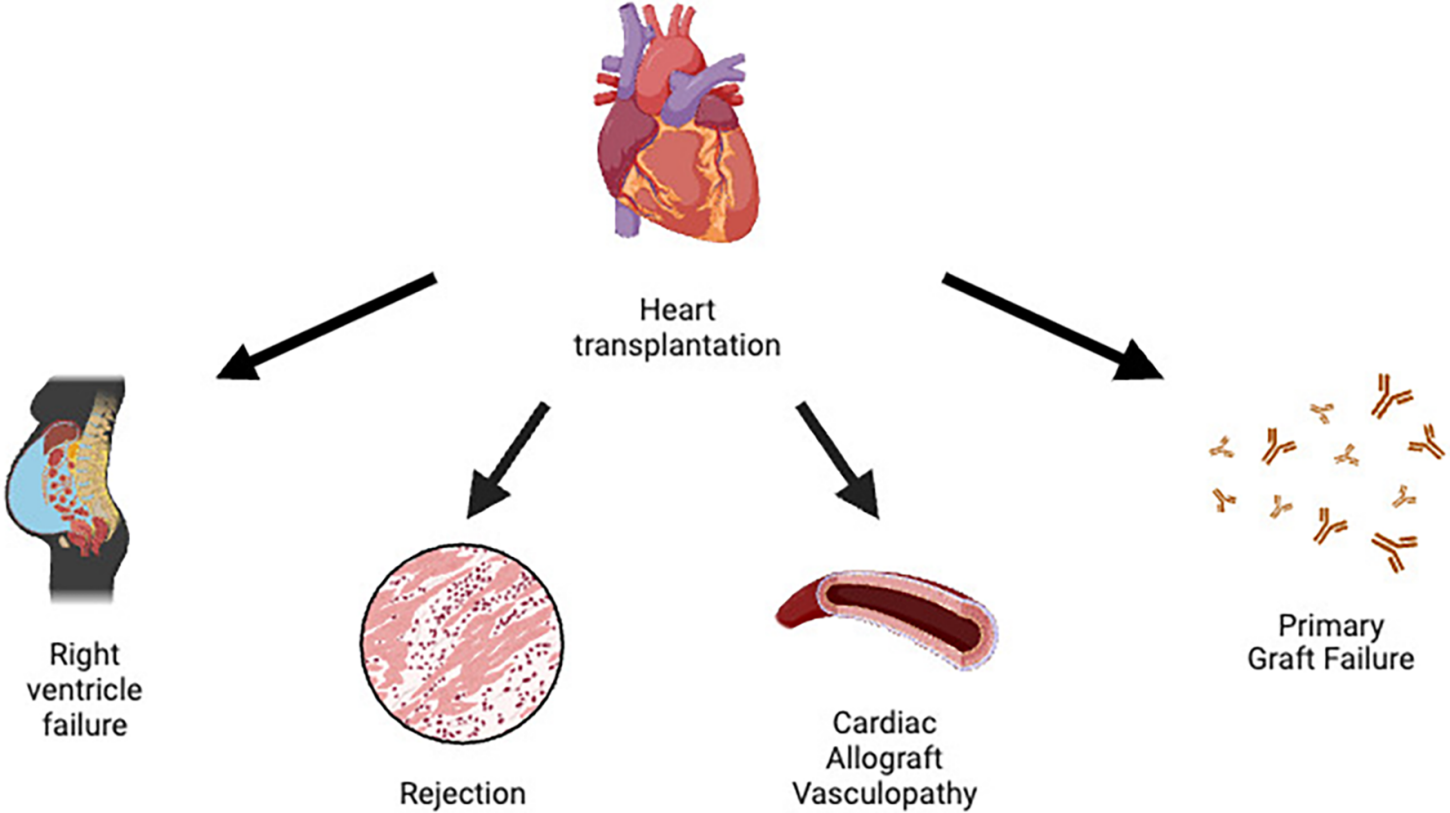
Main heart transplantation complications.

A summary of HTx complications can be found in Figure 1.

### Natriuretic peptides

The International Program on Chemical Safety (IPCS), led by the World Health Organization (WHO), describes biomarkers as “any substance, structure or process that can be measured in the body or its products and that affects or predicts the occurrence of outcomes or disease” (6).

Natriuretic peptides are a family of hormones/paracrine factors normally secreted by the ventricles of the heart. Atrial natriuretic peptide (ANP) is secreted by the atria, while brain natriuretic peptide (BNP) is secreted by the atria in the physiological state and by the ventricles during left ventricular remodelling. The increasing dilation of the ventricles triggers the secretion of the peptides. The main receptor of ANP and BNP is the natriuretic peptide receptor-A (NPR-A), which lowers blood pressure by causing natriuresis and diuresis, vasodilation, an increase in endothelial permeability and antagonization of the renin-angiotensin system (RAAS). They inhibit ventricular hypertrophy and ventricular remodelling. C-type natriuretic peptide (CNP) is secreted by the atria, ventricles, kidneys and cartilage cells. Its main receptor is the natriuretic peptide receptor-B (NPR-B), whose activity is closely linked to bone growth (7, 8).

BNP and the N-terminal pro-peptide (NT-proBNP) are often elevated in transplanted patients, especially in the first two months after HTx. Several studies have demonstrated the association between elevated BNP blood levels and graft rejection. Ogawa et al. found a close link with cellular rejection. Similarly, Wu et al. demonstrated a correlation between BNP and humoral rejection (9). Mandeep et al. found a connection between hemodynamic disturbances, right-sided cardiac abnormalities (including RVD and tricuspid regurgitation) and elevated BNP levels. They discovered that the hemodynamic disturbances in these patients lead to coronary vasculopathy (10). BNP is also associated with ischemia-reperfusion injury in the early stages after HTx, indeed, Mcllroy et al. found a relation with this and PGD, with a sensitivity of 100% and a specificity of 44% (11).

NT-proBNP was the central molecule in the study led by Arora et al. Using a population of 220 HTx patients, they demonstrated not only a link between the pro-peptide and allograft rejection, CAV and mortality, but also that NT-proBNP can be used to diagnose rejection (12). Avello et al. confirmed the link between this molecule and rejection, determining a sensitivity of 87.6% and a specificity of 70% (13), meanwhile Knook et al. has estimated a cut-off of 63 pmol/L as a risk factor for late rejection > 2R, finding a sensitivity and specificity of 86% and 94% respectively (14). This molecule can even predict the risk of developing CAV prior to HTx: the higher the levels, the higher the likelihood. In addition, the increase in NT-proBNP levels is associated with kidney and liver dysfunction after transplantation (15).

### Troponins

In human heart, troponin complex is a crucial component of muscular fibres, and it consists of three subunits: C, T and I troponin. The last two are currently used to detect myocardial damage and death (16). High-sensitivity troponin T (hs troponin-T) is a biomarker strictly related with PDG, it is indeed higher in patients with such complication, being more specific than NT-proBNP, with high values in the early phases of HTx. Méndez et al. has demonstrated that hs troponin-T can be used for both left and right ventricular graft dysfunction with a sensitivity of 75% and specificity of 87% (17).

Troponin T and I can predict the risk of development of CAV. Also, troponin-T concentrations are significantly associated with microvascular fibrin deposits, arteriolar endothelial activation, macrophage infiltrates, depletion of vascular fibrinolytic and anticoagulant components, which lead to coronary disease (18). On the other hand, Laberrere et al. found that troponin-I is frequently elevated in patients with fibrin deposits in microcirculation (19). Moreover, Miller et al. developed a retrospective analysis which found that modest elevations in donor troponin T and I at the time of cardiac harvest are associated with a reduced long-term risk of CAV (20).

Recent studies have found a strong relationship between hs-troponins and HTx-related problems. Dyer et al. verified their association with rejection in a 42-patient trial in 2012, with an area under the curve (AUC) of 0.89 (21). Fitzsimons et al. demonstrated a sensitivity of 8%–100% and specificity of 13%–88% for ACR detection, with a low PPV and a relatively high NPV (79%–100%) (22), and Patel et al. demonstrated that a cut-off of 15 ng/L had a sensitivity of 94%, specificity 60%, PPV 18%, and NPV 99% (23).

### Soluble suppression of tumorigenesis-2 (sST2)

Interleukin-33 (IL-33), also known as Suppression of Tumorigenesis-2 Ligand (ST2l), is a molecule whose signalling pathway (mediated by its receptor ST2) is strictly associated with the immune system, but which also has anti-apoptotic, anti-fibrotic and anti-hypertrophic effects in the heart (24). In 2002, Weinberg et al. discovered sST2, a decoy receptor for ST2 that is usually overexpressed in myocardial injury or cell death. It is secreted by cardiac myocytes, endothelial cells, alveolar epithelium and various types of blood cells (CD4+ T lymphocytes, mast cells) (25).

Due to its role in myocardial damage, several studies investigated sST2 as a possible marker for allograft rejection. Pascal-Figal et al. reported a correlation between ST2 levels and acute allograft rejection in adults (26), while Matheus et al. confirmed this for the paediatric population (27). Grupper et al. also demonstrated that postoperative sST2 levels ≥35 ng/ml are frequently associated with humoral rejection (28). On the other hand, a 2021 study guided by Zhang, found that low levels of this molecule are related with CAV (29).

### Pro-inflammatory cytokines

Cytokines are molecular mediators which modulate inflammatory response via complex pathways. They are used as biomarkers for many pathologies such as immunoproliferative disorders, inflammatory diseases, sepsis, and heart failure (30).

This family of molecule is usually divided into pro-inflammatory cytokines, such as Tumour Necrosis Factor- α (TNF-α), Interleukin-1β (IL-1 β), IL-6, IL-8 and IL-12, and anti-inflammatory cytokines, IL-4, IL-6, IL-10, IL-11, IL-13, IL-1 receptor antagonist (IL-1RA) and TGF-β. While the first group facilitates inflammatory reactions, communicating with the surrounding tissues of a death cell and entering the systemic circulation to activate the immune cells and to induce the acute-phase reaction, the second group inhibits inflammatory pathways and the production of pro-inflammatory cytokines (31).

While the first group promotes inflammatory responses by communicating with the surrounding tissue of a dead cell and entering the systemic circulation to activate immune cells and trigger the acute-phase response, the second group inhibits inflammatory pathways and the production of pro-inflammatory cytokines (31).

Deng et al. have shown an association, in the early post-operative period, between raised IL-6 and TNF- α levels, IL-2 suppression and early systolic and diastolic dysfunction. Even more, IL-2 suppression was related to acute rejection (32). Similar findings were confirmed for the right ventricle by Lei (33).

Przybylek et al. investigated several molecules that could be used as a CAV biomarker. They found a positive correlation between pro-inflammatory cytokines (except for interferon-*γ*) and coronary vasculopathy. These findings were also confirmed for the right ventricle (34).

### Donor specific antibodies

Anti-HLA donor-specific antibodies (DSA) develop in 50% of solid organ transplant recipients (35). Antibodies like these are seen in 3%–11% of HTx patients prior to treatment, and 10%–30% acquire DSA after transplantation (*de novo* DSA) (36). Immune-assay is used to evaluate them, and the most recent technique, the Scintillation Proximity Assay (SPA), has outperformed the Complement-Dependent Cytotoxicity (CDC) assay in both sensitivity and specificity. SPA tests the specificity and relative strength of complement-binding and non-complement-binding HLA antibodies by attaching single pure HLA antigens to distinct microspheres (36).

Anti-HLA DSA are found in almost every humoral rejection after HTx, so they were removed from the ISHLT criteria for humoral rejection in 2012 (although their evaluation is still strongly recommended) (35, 36). Studies have demonstrated their association with both asymptomatic and symptomatic CAV, and have even underlined their presence as a risk factor for coronary vasculopathy (36). DSA against anti-HLA class II, especially DQ, are most strongly associated with graft loss (36).

There are specific DSA antibodies that can be used to detect HTx complications. Anti-MHC class I related chain A (MICA) detect cellular rejection (37), while the presence of anti-angiotensin 1 receptor (AT1R) prior to transplantation increases the risk of cellular and humoral rejection (38). Anti-vimentin antibodies are usually associated with the development of CAV (39).

Tran et al. (2016) investigated the formation of *de novo* DSA antibodies and CAV, rejection, and graft survival in paediatric HTx patients, discovering a significant adverse impact in all of them (40). Previously, in 2008, Kaczmarek et al. identified the same high connections with adult patients in a trial with 213 HTx patients (41), while Smith et al. and Ho et al. recognized *de novo* DSA as a predictor of poor survival (42, 43).

In terms of humoral rejection and CAV, Nath et al. revealed a link between them and DSA antibodies in 2010 (44), conclusions that were corroborated by Ho et al. in adult patients (45) and Ware et al. and Peng et al. in children (46, 47). Several more trials confirmed this relationship and found a robust correlation between CAV and humoral rejection in these patients (48–50).

### Circulating extracellular vescicles

The Extracellular Vesicles (EVs) are lipidic vesicles secreted by all types of cells into the extracellular matrix. They are divided according to their size, biochemistry, and function in micro vesicles, exosomes and apoptotic bodies. Castellani et al. developed a study which demonstrated the higher number of EVs concentration in plasma in patients with cellular or humoral rejection with a receiver operating characteristics curves provided an AUC range of 0.73–0.94 for detecting acute rejection and an accuracy of 86.5% (51). Kennel et al. in 2018 identified 15 differentially expressed proteins during rejection using liquid chromatography–tandem mass spectrometry analysis of serum exosomes (52). Vesicles C4d + are the most common studied and are linked with humoral rejection (53), indeed Hu et al. in 2020, after the purification of donors' heart exosomes with anti-donor HLA I antibody beads, identified C4d protein expression as marker for acute rejection (54).

### Donor derived cell-free DNA

Cell-free DNA (cfDNA) is circulating DNA generated from dead cells; in transplanted patients, cfDNA may originate from both the recipient and the donor of the graft, in which case it is known as donor-derived cfDNA (dd-cfDNA). It is derived from apoptotic and necrotic cells and can be utilized to detect rejection. Recent methods to differentiate recipient cfDNA from dd-cfDNA include single nucleotide polymorphisms (SNPs), genotyping the whole-genome using the patients' pre-transplant DNA, and then comparing each donor-recipient pair to identify meaningful SNPs. A different technique is to estimate the recipient genotype, and then use a computational approach to detect donor and recipient SNPs (55).

Multiple investigations have demonstrated that elevated dd-cfDNA in patients typically correlates with CAV, acute rejection, and poor survival (56–58). Snyder et al. found a rise of dd-cfDNA in the setting of acute rejection with an AUC of 0.84 at a threshold of 1.7% for the detection of 2R acute cellular rejection (ACR) or humoral rejection (AMR) in 2011 (59), Vlaminck et al. found similar results in 2014, with an AUC of 0.83 and 0.95 for the diagnosis of moderate and severe rejection, respectively (60). Agbor-Enoh et al. observed that patients with histological evidence of acute rejection had higher dd-cfDNA levels than controls with mild or no rejection (0.38% vs. 0.03%, *p* 0.001), and that levels were higher in AMR than ACR (61). Finally, the multicentre trial from AlloMap Registry validated the clinical utility of dd-cfDNA for acute allograft rejection surveillance, finding a NPV of 97% and sensitivity of 44% (62).

### Gene expression profiling

Measuring the expression of genes in different cell types is known as gene expression profiling (GEP). This needs to be ascertained by evaluating the cellular quantities of messenger RNA (mRNA) utilizing real-time PCR (rt-PCR) and next-generation sequencing technologies. Transcription is the process by which mRNA is produced from DNA, and it is often utilized as a building block in the endoplasmic reticulum for protein synthesis (63).

Using rt-PCR, the Cardiac Allograft Rejection Gene Expression Observational (CARGO) investigation assessed 11 genes in peripheral blood mononuclear cells. In stable HTx patients, the overexpression of these genes may be utilized to identify moderate-to-severe acute cellular rejection. With a 99% NPV and a 10% PPV, the AlloMap test is the commercial molecular expression of GEP (64). The use of this test in patients with a low pre-test chance of rejection has been confirmed by the HEARTBiT research and other studies (65), providing a NPV of 99% for moderate-severe cellular rejection and low PPV (66–68).

Tarazon et al. have recently investigated the expression of mitochondrial genes in peripheral blood cells as a potential rejection diagnostic (69), moreover, these RNA sequences have been shown to activate the immune system, which makes them both a possible rejection mediator and a marker (70, 71).

The Invasive Monitoring Attenuation through Gene Expression (IMAGE) study noticed that the 2-year cumulative rates for composite primary outcome patients monitored with AlloMap and EMB were similar, 14.5% and 15.3%, verifying that HTx patients with low rejection risk who received heart transplant for more than 6 months can benefit from PBMC GEP instead of invasive assessment (72). The CARGO II trial confirmed its predecessor's outcomes, achieving PPV values of 4% and 4.3% at months 2–6 and >6 after HTx, respectively, and NPV values of 98.4% and 98.3% (66). Finally, Loupy et al. revealed that tissue-based evaluations of multiple pathogenesis-based transcripts suggesting NK burden, endothelium activation, macrophage burden, and interferon-effects can be utilized to identify humoral rejection and correlate with the degree of injury and disease activity (73). while Shahzad et al. conducted a pilot study on the capacity of PBMC GEP to evaluate CAV with good results in 2010 (74).

### Micro RNA

MicroRNAs, or non-coding RNA sequences of 20–25 nucleotides, serve a variety of purposes in human cells. In order to inhibit or enhance gene transcription, they are frequently attached to mRNA or proteins (75). Different types of miRNAs are released by death cells in response to different types of allograft rejection (76).

Three different types of miRNAs—miR-139-5p, miR-151a-5p, and miR-186-5p—that were enhanced in HTx rejection have recently been found by Kennel et al. MiR-29c-3p was discovered to be typically linked to humoral rejection, whereas miR-486-5p was linked to cellular rejection (despite being highly expressed in red blood cells, which may also have high levels in haemolysis) (77, 78).

Conversely, an increase in endothelium-enriched miRNAs is typically associated with CAV. According to Singh et al., individuals with CAV had increased levels of miR-21-5p, miR-92a-3p, miR-92a1-5p, miR-126-3p, and miR-126-5p; miR-92a-3p was especially elevated in patients with stable atherosclerosis, and miR-92a-1-5p levels in native CAV (79). MiR-628-5p was effectively analysed by Neumann et al. as a novel CAV marker (80).

Despite the fact that numerous investigations in the general community have been designed, the connection between miRNAs and diastolic functioning in the HTx population has not been thoroughly examined. In particular, Chade et al. found high levels of miR-183 and miR-376c and low concentrations of miR-1271-3p and miR-196a in diastolic cardiomyopathy (81), meanwhile Zhang et al. demonstrated the positive correlation between miR-19b-3p and miR-181b-5p and diastolic dysfunction (82) and Sun et al. had the same results with miR-133a-1-rs8089787 (83).

### Other biomarkers

A molecule known as antigen carbohydrate-125 (CA-125) is frequently utilized in oncology as a marker for endometrial, gallbladder, and ovarian cancer (84–86). It often drops in the first few months following transplant in HTx patients, typically by 10–20 UI/mL eight months following surgery. According to López-Vilella et al., cellular rejection was linked to its rise (87).

Drugs intended to prevent rejection often have antiproliferative effects, which result in lymphocytopenia, which is defined as a lymphocyte count <3,000/mm^3^ in HTx patients. Intriguingly, a number of investigations revealed a correlation between temporary lymphocytopenia and CAV, autoimmune responses, and rejection. This is most likely due to the relationship between the condition and the reactive buildup of donor T-cells, which compromises peripheral tolerance (88). The lymphocyte-neutrophil ratio (LNR), which has been shown by Choi et al. to be a significant HTx producer and that levels >0.46 are linked to acute rejection (89).

Finally, as an indicator of inflammation, the liver and adipose tissue release C Reactive Protein (CRP), an acute phase protein. In HTx patients, Arora et al. discovered both a correlation between CRP, CAV, and CRP and CRP and all-cause death (12).

**Figure 2:**
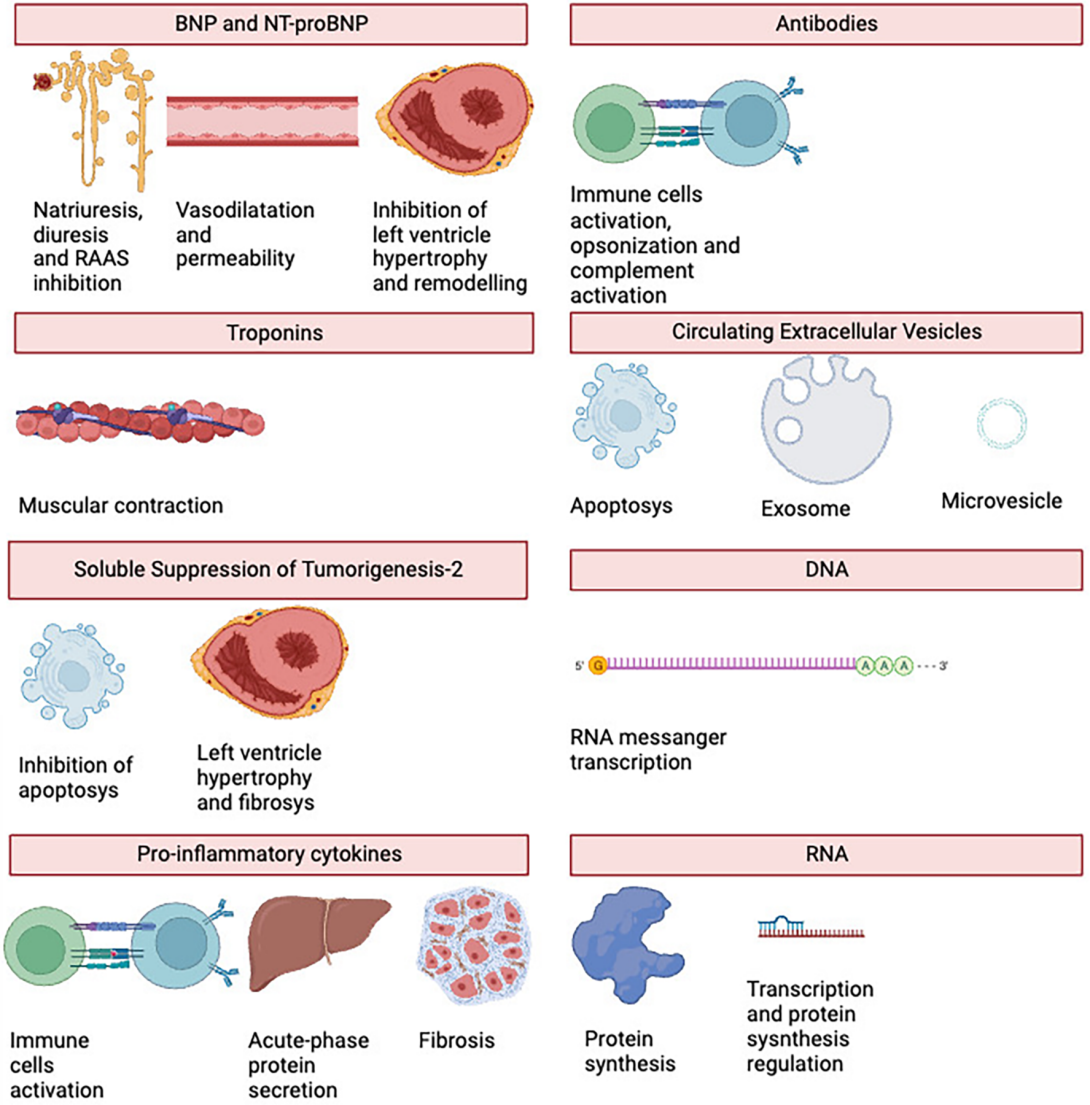
Main biomarkers in heart transplantation and their principal activities.

See Figure 2 for an overview of the biomarkers' primary functions, and Table 3 for a table showing the relationships between the biomarkers and HTx problems.

**Table 3:** Synthesis of HTx complications and biomarkers association, ordered by their sensitivity and specificity.

Complication	Sensitivity	Specificity
Rejection	• BNP (+++) • NT-proBNP (+++) • sST2 (++) • DSA (++) • dd-cfDNA (++) • GEP (++) • miR 139-5p, miR 151a-5p, miR 186-5p (++) • EV (+) • CA 125 (+) • Lymphocytopenia (+) • Low LNR (+)	• miR 139-5p, miR 151a-5p, miR 186-5p (+++) • sST2 (+) • BNP (+) • NT-proBNP (+) • DSA (+) • dd-cfDNA (+) • GEP (+) • EV (+) • CA 125 (+) • Lymphocytopenia (+) • Low LNR (+)
CAV	• miR 21-5p, miR-92a-3p, miR-92a1-5p, miR-126-3p, miR-126-5p (+++) • NT-proBNP (+++) • hs-troponins (+++) • DSA (++) • CRP (++) • EV (+) • Pro-inflammatory cytokines (+)	• miR 21-5p, miR-92a-3p, miR-92a1-5p, miR-126-3p, miR-126-5p (+++) • NT-proBNP (+) • hs-troponins (+) • Pro-inflammatory cytokines (+) • DSA (+) • EV (+) • CRP (+)
PGD	• BNP (+++) • Pro-inflammatory cytokines (++)	• BNP (+) • Pro-inflammatory cytokines (+)
RVD	• BNP (+++) • hs-troponins (+++) • Pro-inflammatory cytokines (+)	• BNP (+) • hs-troponins (+) • Pro-inflammatory cytokines (+)
Mortality	• NT-proBNP (+++) • CRP (++)	• NT-proBNP (+) • CRP (+)

### Biomarkers in the early post transplant period

In addition to PGD, during the first month after the HTx the most common complications are arrhythmias and pericardial effusion. Less common problems are mediastinal haemorrhage and sternal wound infection (90).

Arrhythmias in transplanted patients is commonly in case of prolonged graft ischemia, perioperative reperfusion injury, re-warming of cold myocardial tissue, blunt trauma, and unbalanced serum electrolytes (91). Furthermore, a time of ischemia superior to 4 h increases 30-days, 1-year mortality and the rates of chronic rejection (92). Sympathetic denervation, surgical trauma, ischemic injury to the sinus node, and graft ischemia are the main causes of post-surgical bradycardia, which is one of the most common arrhythmias in the early-postoperative (93). Atrial fibrillation, on the other hand, is one of the most common tachyarrhythmias, being detected in 10%–24% of HTx patients (94). Finally, premature ventricular contractions are present in 100% of these patients in the first month after HTx and are not associated with mortality (95), but worsening or sustained ventricular arrhythmia must be investigated immediately since their association with CAV and acute rejection (92, 96–98).

Albert et al. noticed that high CRP levels were associated with an increased risk of SCD, whereas Streitner et al. reported high levels of CRP, NT-proBNP, and IL-6 in patients who experienced an electrical storm (99, 100). IL-6 is closely linked to ventricular tachycardia (VT), ventricular fibrillation (VF), and SCD (101–103). Several studies have shown a connection between high levels of natriuretic peptides and hs-troponins and VF/VT and SCD(104–108). Liu et al., in particular, hypothesized an ischemic explanation for cardiac arrhythmias, citing troponins generated during myocardial necrosis (109). The MADIT-CRT trial reported an increased risk of ventricular arrhythmias and death in patients with a 10% increase in sST2 concentration after one year, although an elevated concentration at baseline was not predictive of VT/VF (110). Finally, higher levels of Galectin-3, a protein expressed after tissue damage that is involved in mRNA splicing, anti-apoptotic signalling control, pathogen detection, and inflammation processes, can predict ventricular arrhythmias in SCD patients (111).

Pericardial effusion is often found after HTx, especially in patients with no prior cardiac surgery (112, 113), in cases of receiver-donor mismatch (113), and in patients receiving cyclosporine as part of immunosuppressive medication (114). It usually occurs within the first three months of surgery, and its relationship with graft rejection is controversial; Valentine et al. and Ciliberto et al. saw a positive correlation between them (115), Hauptman et al., on the other hand, noticed a lack of association between acute rejection and pericardial effusion (113). Tanaka et al. and Kramer et al. reported that high BNP levels in pericardial fluid (PF) are connected to both systolic and diastolic left ventricular dysfunction (116, 117), whereas fibroblast growth factor 2 levels are linked to the extent of coronary collaterals (118, 119) and unstable angina (120). Finally, fatty-acid binding protein and troponins higher in PF than plasma are correlated with myocardial ischemia (121–123).

### Future perspectives

Every day, new biomarkers are identified and new technologies are applied in laboratories to render identification more accurate and sensible. The newly discovered molecules can be employed for a variety of purposes, including recognizing high-risk patients, determining pathologies' specific phenotypes, and analysing specific therapeutic targets.

In regard to the first functionality, predicting the risk as early as feasible and establishing risk ratings can assist the physician in determining when and how to begin the appropriate therapy. Genomic sequencing and nucleic acid-based biomarkers (miRNAs, cfDNA, etc.) may hold the key for accomplishing this objective. Furthermore, these new biomarkers might identify individual resistances and vulnerability to diseases, as well as their clinical manifestations, allowing for highly specific precision treatment. Finally, the distinct interactions with a drug could be determined, enabling not only to initiate more appropriate immunosuppressive therapies to reduce the incidences of HTx comorbidities, but also to predict potential adverse effects and pharmacokinetic properties even before the drug was assumed.

## Conclusions

Many biomarkers are available to aid in the early detection of cardiovascular disease in recipients of heart transplants. These indicators can be used in conjunction with contemporary imaging techniques like T1/T2-mapping in cardiac magnetic resonance and Speckle Tracking Echocardiography to identify early cardiac dysfunction and ensure that patients receiving the best care possible on time. The primary drawback of the biomarkers examined in HTx is their frequent lack of specificity; for example, hs-troponins may be raised in both CAV and PDG. dd-cfDNA is not useful for diagnosing patients who have had numerous transplants. RNA molecules are scarcer, but new research indicates that they are more selective.
